# Arsenic in Slovakia: Pollution Issues and the Potential of Magnetic Carbon Biomaterials for Wastewater Treatment

**DOI:** 10.3390/ma18020289

**Published:** 2025-01-10

**Authors:** Anton Zubrik, Eva Mačingová, Slavomír Hredzák, Marek Matik

**Affiliations:** Institute of Geotechnics, Slovak Academy of Sciences, Watsonova 45, 040 01 Košice, Slovakia; macingova@saske.sk (E.M.); hredzak@saske.sk (S.H.); matik@saske.sk (M.M.)

**Keywords:** arsenic contamination, real wastewater, remediation, magnetic carbon bioadsorbents

## Abstract

In Slovakia, there are a number of contaminated sites that have occurred due to intensive mining, mineral processing, metallurgical activities, chemical industry, fossil fuel combustion, and industrial agriculture in the past. This paper summarizes the occurrence, chemistry, toxicity, and mineralogy of arsenic species related to soil and water contamination in Slovakia. Four main localities with arsenic exposure were identified. Additionally, magnetically modified carbon biochar (MWchar-Mag) was tested for arsenic removal from a model solution alongside real mine water discharged from the abandoned Hauser adit. For the model aqueous solution, the maximum adsorption capacity was established at 6.2 mg of As per g of MWchar-Mag at natural pH. In the case of mine water with a concentration of arsenic at around 0.2 mg/L, adsorbent dosage, pH influence experiments, and kinetic tests were realized. The results confirmed 100% arsenic removal efficiency at pH~3–7 and extremely fast kinetics.

## 1. Introduction

### 1.1. Review on Arsenic Toxicity, Chemistry, Mineralogy and Industrial Application

Contaminated drinking water is a major source of arsenic poisoning worldwide. Arsenic can also enter the human body through food (vegetables grown in contaminated soil), smoke, cosmetics, and air (industrial areas). Arsenic is a dangerous carcinogenic contaminant. Long-term exposure can cause serious illnesses, including cancers, diabetes, cardiovascular disorders, melanosis, hyperkeratosis, and peripheral neuropathy. Chung et al. [1] described the various potential pathways of arsenic exposure affecting the human body. The World Health Organization, as well as Slovak legislation (Decree MoH SR No 247/2017 Coll.), set a maximum arsenic concentration of 0.01 mg/L in drinking water.

Arsenic is a chemical element classified in group 15 of the periodic table (atomic number—33; atomic mass ~ 75). Arsenic occurs naturally as the isotope ^75^As and primarily exists in three oxidation states (−3, +3, and +5) [2]. Organically bonded arsenic (methylated arsenic) is generally less toxic than inorganic arsenic species. The primary inorganic forms of arsenic in water are As^3+^ and As^5+^ [3]. The As⁵^+^ ion dissociates in aqueous solutions, forming oxyanions (pK = 2.2, 7.0, 11.5) as follows: pH < 2.2—H_3_AsO_4_^0^; pH 2.2–7.0—H_2_AsO_4_^−^; pH 7.0–11.5—HAsO_4_^2−^ and pH > 11.5—AsO_4_^3−^. For As^3+^, the pK values are 9.2, 12.1, 13.4 (pH < 9.2—H_3_AsO_3_^0^; pH 9.2–12.1—H_2_AsO_3_^1−^; pH 12.1–13.4—HAsO_3_^2−^; pH > 13.4—AsO_3_^3−^). Trivalent arsenic exhibits greater mobility and toxicity than pentavalent arsenic. Compared to the negatively charged As^5+^, As^3+^ exists as a neutral molecule under natural conditions (up to pH 9.2). Thus, its affinity for mineral surfaces and other natural materials is weaker. As^3+^ is more common in groundwater under anaerobic conditions and can rapidly oxidize to arsenate species. In contrast, As^5+^ predominates in surface water. The redox potential and pH are the key factors influencing arsenic speciation [4,5].

Arsenic ranks as the 20th most common element in the earth’s crust. The terrestrial abundance of arsenic is approximately 5 mg/kg [6,7]. The Geochemical Atlas of Europe [2] demonstrates that the total crustal abundance of arsenic is 2.5 mg/kg and 4.8 mg/kg in the upper crust [8], while McLennan and Taylor [9] report average bulk crustal abundances of 1.0 mg/kg and 1.5 mg/kg for the upper continental crust. Inorganic arsenic is present in more than 200 mineral species [6,7]. Arsenic is strongly chalcophile and is primarily partitioned into a variety of sulfide and sulfarsenide minerals, primarily arsenopyrite FeAsS, realgar AsS (or As_4_S_4_), orpiment As_2_S_3_, tennantite Cu_11_Fe^2+^As_4_S_13_ (or Cu_6_[Cu_4_(Fe,Zn)_2_]As_4_S_13_), minor alacránite As_8_S_9_, uzonite As_4_S_5_, and enargite Cu_3_AsS_4_ (orthorhombic), luzonite Cu_3_AsS_4_ (tetragonal), gersdorffite NiAsS, cobaltite CoAsS, glaucodot (Co,Fe)AsS (or Co_0.75_Fe^2+^_0.25_AsS, skutterudite (Co,Ni)As_3−x_, (or Co_0.75_Ni_0.25_As_2.5_, CoAs_2−3_), safflorite (Co,Fe)As_2_, (or Co_0.75_Fe^2+^_0.25_As_2_), rammelsbergite NiAs_2_, loellingite FeAs_2_, nickeline NiAs, langisite (Co,Ni)As (or Co_0.75_Ni_0.25_As), maucherite Ni_11_As_8_, and sperrylite PtAs_2_. Significant As secondary minerals are arsenates such as scorodite Fe^3+^(AsO_4_)·2(H_2_O), annabergite Ni_3_(AsO_4_)_2_·8(H_2_O), erythrite Co_3_(AsO_4_)_2_·8(H_2_O), mansfieldite AlAsO_4_·2(H_2_O) and oxides, e.g., arsenolite As_2_O_3_, as a product of alteration [2,10,11,12]. Arsenic can occur as native element arsenic As (trigonal), arsenolamprite As (orthorhombic), and pararsenolamprite As or As_0.94_Sb_0.05_S_0.01_ (orthorhombic). Arsenic is also widely present as an accessory element in other sulfide minerals such as pyrite FeS_2_ (e.g., 119 mg/kg of As in pyrite from the Slovak talc deposit at the Gemerská Poloma, the Spiš-Gemer Ore Mts. [13]), galena PbS, and sphalerite ZnS [2]. Arsenic occurs in several types of deposits, mainly stratiform Cu–PGE–Ni deposits in the basal zone of (ultra)basic complexes, As in porphyry Cu deposits including co-magmatic breccia pipes, As in Sn skarn deposits, unconformity-related realgar and orpiment deposits, orogenic As–Au veins, As–Tl–Pb–Cu–Ag deposits in dolomitic metacarbonates, and As can also be present in coal. Arsenic is primarily recovered as a by-product of ores containing arsenopyrite, loellingite, realgar, or orpiment or as a minor constituent of Sn–W–, Cu–(Ni), Pb–, Au–, Co–(Ni), and pyrite ore [12,14].

Despite their toxicity, arsenic compounds are widely used in industries. High-purity arsenic (99.999%) is applied to produce gallium-arsenide (GaAs) and indium-arsenide (InAs) semiconductors for solar cells, computers, electronics, space research, and telecommunications, and also for germanium-arsenide–selenide (GeAsSe) specialty optical materials. Indium–gallium-arsenide (InGaAs) is employed in short-wave infrared technology [14]. Arsine (AsH_3_) is used as a doping element for the production of crystals (computer chips and fiber optics) [15]. Lead–acid battery grids are strengthened by an admixture of arsenic metalloids. Arsenic is also used as an antifriction additive for bearings to harden lead shots and clip-on wheel weights [14], as well as in automotive solders and radiators [15]. The addition of very small quantities of arsenic to copper enhances corrosion resistance. It prevents cracking in brass [16]. Arsenic compounds are used in herbicides and insecticides [14] and also in pigments, sheep dips, leather preservatives, poisonous baits, catalysts, pyrotechnics, antifouling agents in paints, pharmaceutical substances, ceramics, dyes, and soaps [15]. According to George [14], arsenic trioxide is used for arsenic acid, and subsequently, chromated copper-arsenide preservatives are produced for pressure-treating lumber applied primarily in non-residential spaces. The annual production of arsenic reached approximately 56.4–56.9 kilotons in 2022. The major producers are Peru 25.379 kt (45.0%), China 24 kt (42.55%), and Morocco 5.45 kt (9.66%), i.e., together they produce more than 97% of the annual world production [17,18]. Thus, As contamination is often caused by mining, mineral processing, and smelting. Li et al. [19] referred to the heavy metal loading of soil in mining areas in China (72 mines were evaluated). The mean concentration values of Cd, Pb, Cu, Zn, Hg, As, and Ni were compared with soil in mining areas around the world (Vietnam, India, Spain, South Korea, and Iran). The arsenic concentration varies from 19 mg/kg to 3144 mg/kg. Besedin et al. [20] summarized As content in mine waste and mine-impacted soil in selected gold mining areas in the world. As concentration can attain up to 98,300 mg/kg in mine waste (New Zealand) and 12,483 mg/kg in soil (USA), respectively. The source of contamination is often arsenopyrite and/or As-bearing pyrite, which host gold in their structure as a common impurity.

### 1.2. Natural Occurrence in Slovakia

In Slovakia, arsenic contamination is a significant problem due to the rich mining and industrial history and its natural occurrence. The average contents of arsenic in Slovakian rocks vary from 1.1 to 1.5 mg/kg in Neogene volcanics to 7.4 mg/kg in claystones. The highest As content, 11.8 mg/kg, was detected in greisenized Gemeric granite due to subsequent hydrothermal processes [21]. Arsenic is further transported via waterways to stream sediments and soil. The average As content in Slovakian stream sediments is about 11 mg/kg. The highest anomalies from 10 to 93 mg/kg occur in the Spiš-Gemer Ore Mts. (the presence of sulphidic metal ore deposits and mineralization occurrence—mean 49.2 mg/kg and median 16.60 mg/kg—are referred to for areas of the Early Paleozoic of Gemericum) and in the basin of the Slaná, the Hornád and the Bodva rivers, which drain this area. The enhanced values of arsenic were detected in the crystalline complexes of the Low Tatras Mts. (Central Slovakia) and the Little Carpathians Mts. (Western Slovakia) (mean 24.59 mg/kg, median 9.63 mg/kg). High arsenic content was also determined in Neogene volcanics (mean 8.31 mg/kg and median 5.04 mg/kg), mainly in the Štiavnica Mountains (Štiavnické vrchy in Slovak) and the Kremnica Mountains (Kremnické vrchy in Slovak), both in Southern Central Slovakia where sulfide metal ore deposits and mineralization occur.

In total, 10,035 Slovak soil samples were assayed to determine their total arsenic contents [22]. The arsenic contents vary in the range of 0.1–2500 mg/kg with a significant interval of 0.5–10.4 mg/kg and a median value of 7.5 mg/kg. A high correlation of As contents was detected between As and Sb in lithosol and phaeozem, as well as a high correlation in chernozem. Similarly, a high correlation was found between As and Hg in regosol, and finally, the contents of As highly correlated with the contents of Cu, Hg, and Pb in phaeozem. In the case of Slovak groundwater, 16,359 samples were collected from springs, drill holes, adits, wells, and dewatering systems and were assayed. The arsenic mean concentration was 1.9 μg/L, and the median was 0.5 μg/L. Enhanced values of As contents were caused by two sources firstly, the occurrence of sulfide ore deposits, namely in the Spiš-Gemer Ore Mts., the Low Tatras Mts., the Little Carpathians Mts., and the Neogene volcanics. The second—anthropogenic pollution—may attain up to 50–250 μg/L [23].

### 1.3. Anthropogenic Pollution in Slovakia from Ore Mining and Processing

Historical mining, coal combustion, and the chemical industry are the major anthropogenic contributors to arsenic contamination in Slovakia. The abandoned Dúbrava Sb(–Au) deposit in the Low Tatras Mts. (Central Slovakia) represents the most significant threat to Sb pollution in Slovakia and is presumably one of the weightiest deposits globally. The main source of contamination is the discharge from old mine adits. The outflow from the Samuel adit exhibited the highest content of Sb and As, reaching 9300 µg/L and 62 µg/L, respectively. High concentrations of Sb (104 µg/L) and As (16 µg/L) were documented in seepage waters originating from the tailings area. In the Paludžanka Creek alluvium downstream from the tailings, Sb and As concentrations reached maximum levels of 9616 mg/kg and 844 mg/kg, respectively [24]. Medzibrod, located in the southern part of the Low Tatras Mts., is another territory affected by contamination after Sb–Au ore mining. The waste generated from sulfide ore extraction was deposited in a tailing impoundment situated in the Borovský Brook valley. Stream water below the mines exhibited strongly elevated levels of As and Sb, reaching concentrations of up to 255 µg/L and 1290 µg/L, respectively. Hydrous ferric oxides (HFOs) precipitating from the Murgaš adit showed an arsenic concentration of 206 mg/kg. Stream sediments in the Borovský Creek, near the adit, displayed a high content of As (9180 mg/kg) and Sb (890 mg/kg) [25].

The Pernek Sb(–As–Fe–Au–Ag) deposit, located approximately 3 km from the village of Pernek in the Little Carpathians Mts. (Southwestern Slovakia), was investigated by Hiller et al. [25]. Stream waters from the Kostolný Creek, which drains the mine site, showed low levels of As and Sb, whereas the outflow from waste rock dumps exhibited the highest Sb concentration (31 µg/L). HFO precipitates in downstream waters displaying high concentrations of As (up to 2009 mg/kg) and Sb (up to 2155 mg/kg). The content of As in stream sediments varies between 45 and 390 mg/kg, while Sb concentrations range from 56 to 703 mg/kg. Within the Little Carpathians Mts., the abandoned Sb(–Au–As) deposit Pezinok is another site that represents a significant source of both As and Sb contamination. The sources of the metalloids are two tailing impoundments and several outflows from the adit. In the downstream direction, the impoundments discharge voluminous masses of hydrous ferric oxides locally, with an extremely high As_2_O_5_ and Sb_2_O_5_ content of 28.3 wt% and 2.7 wt%, respectively [26]. Boreholes directly located in the mine tailing ponds exhibited the highest arsenic and antimony concentrations, reaching up to 90,000 µg/L and 7500 µg/L, respectively. Analyses revealed severe groundwater contamination with concentrations reaching up to 27,380 µg/L of As and 7750 µg/L of Sb. The highest arsenic and antimony content in surface water, 104 µg/L and 645 µg/L, respectively, were found in mine water discharged from the Pyritova adit [27].

Several highly contaminated localities are situated in the Spiš-Gemer Ore Mts. (Eastern Slovakia). The Čučma deposit (Au–Ag–Cu ore mined in the Middle Ages, later Sb and Mn ores), located in the southern part, was studied by Hiller et al. [25]. Two streams, the Laz and the Čučmiansky Creeks, drain the area. High Sb and As concentrations were detected in mine waters, reaching up to 3540 µg/L and 1350 µg/L, respectively. Downstream in the Laz Creek, the Sb and As content achieved values of 116 µg/L and 670 µg/L, respectively. Below the confluence with the Čučmiansky Creek, As and Sb concentrations in water decreased, with a more pronounced decline observed for As. Stream sediments exhibited maximum As and Sb concentrations of 187 mg/kg and 600 mg/kg, respectively. The soil at the mine site displayed higher arsenic and antimony content-reaching values of 190 mg/kg for As and 782 mg/kg for Sb. The Poproč Sb deposit can be found in the southeastern region of the Spiš-Gemer Ore Mts. Tailings generated from the flotation of sulfide ores were disposed of three impoundments at this location. The Olšava Creek, draining the mine site, directly receives mine water from abandoned adits and tailing impoundments. Water draining from adits and impoundments exhibited high concentrations of both metalloids, up to 2150 µg/L of As and 750 µg/L of Sb. These mine waters are distinctive by the precipitation of abundant HFO layers, with the concentration of As and Sb reaching 60,412 mg/kg and 17,173 mg/kg, respectively. HFOs play a significant role in the natural attenuation of both metalloids in stream waters, although this is more considerable for As than Sb. Sediments in the Olšava Creek below the adits and mine workings exhibited elevated levels of As and Sb, reaching maximum concentrations of 5560 mg/kg and 1360 mg/kg, respectively [25]. Jurkovič et al. [28] determined the content of As and Sb in highly contaminated soil. The concentration of metalloids was in the range of 28–2484 mg/kg for As and 13.4–5757 mg/kg for Sb. The Zlatá Idka village, located in the eastern part of the Spiš-Gemer Ore Mts., is distinctive for its intensive mining and ore processing operations. Between the 14th and 19th centuries, As-bearing Ag–Au–Sb ores were exploited. Geochemical analyses indicate the high pollution of the surrounding environment by As and Sb. The average As and Sb contents are 892 mg/kg and 818 mg/kg in soil, 195 mg/kg and 249 mg/kg in stream sediments, 28 µg/L and 21 µg/L in groundwater and 24 µg/L and 34 µg/L in surface water [29]. Hredzák et al. [30] studied ochres (HFOs) from Marta adit (former siderite mine at Nižná Slaná, Spiš-Gemer Ore Mts., Eastern Slovakia). They determined the arsenic concentration to be in the the range of 7800–18,400 mg/kg. In all the above-mentioned abandoned metal ore deposits and mines, arsenopyrite FeAsS and/or As–pyrite FeS_2_ are the main sources of arsenic contamination. Both minerals accompany utility minerals such as stibnite Sb_2_S_3_, gold, (Ag–) tetrahedrite Cu_9_Fe^2+^_3_Sb_4_S_13_ or Cu_6_(Cu_4_X^2+^_2_)Sb_4_S_12_S (X^2+^ = Fe^2+^, Zn, Cd, Hg…), (Ag–)jamesonite Pb_4_Fe^2+^Sb_6_S_14_, boulangerite Pb_5_Sb_4_S_11_, siderite FeCO_3_, and chalcopyrite CuFeS_2_, etc. Moreover, arsenopyrite can host gold in such a way that it represents so-called refractory Au ores (Pezinok) [31,32,33,34].

### 1.4. Arsenic Pollution in Relation to Coal Mining, Fossil Fuel Combustion and Chemical Industry

A specific case of an environmental load of arsenic is the deposition of power plant ash as a product of brown coal combustion. During coal burning, most As volatilizes and leaks away to the gas and aerosol phase, with only a minor portion remaining in the bottom ash. The highest amount of the escaping As is captured by fly ash and dispersed to the landscape [35]. A relevant source of an environmental load of arsenic is the deposition of power plant ash, where the primary problem is the mobilization of arsenic into surface- and groundwater. The extractability of arsenic from solid matrices is influenced by the mineralogy, geochemistry, and speciation of As in the lagoons of combustion waste. Moreover, hydrous Fe, Mn, and Al oxides play an important role in the retention of As in natural solids, depending on the pH and redox state of the surroundings [36,37,38]. In Slovakia, there are two localities that have this type of pollution. A significant source of arsenic pollution is located in the district of Prievidza (Nitra Valley, Central Slovakia), where the Nováky Thermal Power Plant burns high-arsenic brown coal from Nováky and Handlová coal deposits [38]. Annual arsenic emissions from power plants were as high as 200 t between 1953 and 1989. Measures executed for pollution control have reduced arsenic emissions to less than 2 t per year [39]. The dominant contamination of this territory was caused by the dam failure of one of the coal–ash ponds in 1965 when 3 million tons of As-rich ash slurry was released into the surrounding environment. Up to 20 km^2^ of mainly agricultural soils was covered by the ash layers with a thickness of 1–2 m. In the frame of remediation, the impacted area was coated by a 30 cm thick layer of soil. In several studies, increased arsenic concentrations in affected soil were documented. Both fresh ash sludge and long-buried power plant ashes contain considerably high concentrations of As, reaching up to 1400 mg/kg^−1^. The average As concentration in the cover soil at a depth of 0–30 cm was 257 mg/kg, and at a depth of 30–60 cm was 411 mg/kg [37,38,40]. An impoundment located near the village of Poša (Eastern Slovakia) is a significant source of arsenic pollution. It had been operated since 1977 by a local chemical factory and contained waste from the chemical industry, deposited burning waste, and coal fly ash. Water leaking from impoundments, enriched in arsenic and other potentially toxic elements, extensively pollute the surface water and stream sediments of watersheds of the Kyjov brook and part of the Ondava River. Extensive field research revealed the widespread arsenic contamination of the aquatic environment in the studied area. The arsenic concentrations in representative sediment and water samples ranged from 36.3 to 3210 mg/kg and 4.05–613 μg/L, respectively [41]. Zinc was also monitored in this area. Concentrations of environmental importance up to 3390 mg/kg were found only in the impoundment materials [42]. Moreover, the extreme concentration of toxic organic compounds (polychlorinated biphenyls) causes serious environmental and health risks in this affected territory.

### 1.5. Arsenic Removal from Water by Magnetic Carbon Biomaterials

There are numerous pollutants, both organic and inorganic in origin, which are non- or poorly biodegradable, requiring natural microbial processes to convert them into non-toxic or at least less-toxic components. Therefore, ongoing efforts focus on enhancing existing remediation technologies and developing innovative materials and techniques for the elimination of these contaminants from the environment [43]. Arsenic, as a highly toxic persistent mobile contaminant, cannot be destroyed, but its adverse environmental impact can be reduced through immobilization processes. Several methods have been developed for arsenic removal from aqueous solutions, including adsorption, precipitation techniques (co-precipitation, chemical precipitation/flocculation), membrane processes (reverse osmosis, (electro)dialysis, nanofiltration), ion exchange, and biological processes. Some of these are commercially available. The cost of the material/technology and its effectiveness in achieving arsenic concentrations below 0.01 mg/L in drinking water are also limiting factors for commercialization.

Adsorption is one of the most effective methods for removing and immobilizing persistent substances from water bodies. In many cases, a key limitation of the adsorption method is the cost of the adsorbent and its limited reusability. The regeneration of the adsorbent, especially using thermal or solvent methods, is often expensive, and the adsorption capacity is significantly lower. Consequently, the adsorbents used are simply discarded as sludge in secure landfills. Thus, preparation for a novel, effective adsorbent with a high adsorption capacity continues to be a subject of considerable research interest. Effective adsorbents require favorable physicochemical surface properties (e.g., an enhanced pore structure and suitable electrochemical properties), facile handling, rapid solid/liquid separation after adsorption, and simple regeneration. Over the past decade, considerable research has focused on developing cost-effective adsorbents derived from abundant natural resources. The literature indicates [43] that adsorption, especially with carbon-based, magnetic sorbents, and biosorbents, offers advantages in terms of its simplicity, broad applicability, high removal efficiency, and cost-effective reusability.

Carbonaceous materials (e.g., carbon sludge, industrial by-products, waste biomass (agricultural and microbial), low-rank coal, and activated carbons and biochars) and natural inorganic materials with developed pore structures (e.g., zeolites and clays) are frequently used for the removal of inorganic (heavy metals/metalloids) and organic pollutants (e.g., PAHs, pesticides, organic dyes, antibiotics, and personal care products). Heavy metals in aquatic environments are predominantly present as cations (e.g., Cd, Pb, Cu, Co, Ni). However, removing highly mobile anionic metalloids such as Cr and As remains challenging, particularly in wastewater containing both anionic and cationic species at different concentrations. In comparison to investigations focused on the adsorption of cationic metal ions, research on arsenic adsorption, especially with low-cost adsorbents, is limited.

The chemical and physical modification of the carbon bioadsorbents can increase adsorption capacity. The adsorption of arsenic can be significantly enhanced through the surface modification of a negatively charged porous carbonaceous matrix and the application of non-toxic inorganic cations (Cu^2+^, Fe^2+^/Fe^3+^). Furthermore, after the adsorption process, the presence of magnetic particles accelerates the solid/liquid separation of the adsorbent from the water solution. For example, Zubrik et al. [44] synthesized magnetic-responsive carbon material from agricultural waste biomass, which was modified by non-stoichiometric maghemite nanoparticles. Magnetic carbon material with homogenously distributed nano-iron oxides incorporated in the porous carbon matrix can effectively adsorb As^5+^ with maximum adsorption capacity (*Q*_m_) at 24.9 mg/g as well as cationic methylene blue dye (*Q*_m_ = 55 mg/g). In the previous paper [45], a rapid microwave-assisted pyrolysis of the mixture of carbon-based biomass/ferrofluid was applied for the synthesis of magnetic biochar. Magnetic biochar showed excellent adsorption properties towards arsenate as well as methylene blue (*Q*_m_(As) = 25.6 mg/g; *Q*_m_(MB) = 144.9 mg/g). Another procedure—non-conventional high-energy ball milling—was used for the preparation of magnetic carbon from coal (Slovak lignite) and ferrofluid [46]. The magnetic coal char was successfully tested as an adsorbent of As^5+^ oxyanions (*Q*_m_ = 19.9 mg/g) and Cd^2+^ cations (*Q*_m_ = 58.8 mg/g). Wen et al. [47] synthesized magnetic porous carbonaceous material via the pyrolysis of tea waste for the efficient removal of As^5+^, Cr^6+^, humic acid, and dyes. The adsorption capacity of the prepared magnetic carbon material for As^5+^ (38.03 mg/g) and Cr^6+^ (21.23 mg/g) was better than commercial bulk Fe_2_O_3_. In the next work, CO_2_ and Fe-modified lignin biochar was produced by the pyrolysis process and tested as an adsorbent of arsenic (V) [48]. It was found that CO_2_ modification caused an increase in the pore volume and surface area of the adsorbent. Nevertheless, the arsenic (V) removal was not effective. The As (V) adsorption efficiency of the biochar increased significantly after modification with FeO_x_ (*Q*_m_ = 6.8 mg/g), although a 50–60% decrease in the BET surface area and porosity was observed. Fan et al. [49] proved that the iron impregnation of corn straw biochar enhances As^5+^ removal. Despite the relatively low surface area value (4.81 m^2^/g), the composite showed a high value of the maximum sorption capacity—*Q*_m_(As) = 17.8 mg/g. The Fe-oxides (magnetite, maghemite, hematite) and cryptocrystalline and/or amorphous iron oxyhydroxide play a crucial role in the adsorption process of As. Similar results were obtained by Cho et al. [50]. A magnetic biochar was prepared by the N_2_- and CO_2_-assisted pyrolysis of spent coffee ground and used as an adsorption medium for As^5+^. The magnetic adsorbent prepared under CO_2_-assisted pyrolysis showed significantly better textural properties (S_BET_(CO_2_) = 512.0 m^2^/g) than the magnetic adsorbent synthesized in a N_2_ atmosphere (S_BET_(N_2_) = 8.3 m^2^/g); however, the sorption properties were almost the same (*Q*_m_(CO_2_) = 12.1 mg/g versus *Q*_m_(N_2_) = 12.6 mg/g). The results showed that the As^5+^ adsorption was governed by the Fe mineral phase composition rather than porosity. The mechanism of arsenic immobilization on Fe biochars prepared by different pyrolysis conditions (*t* = 400–850 °C) was studied in detail by Xu et al. [51]. The authors showed that depending on the pyrolysis temperature, different Fe-phases are formed with a distinct arsenic removal mechanism. Chestnut shell and magnetic gelatin were applied to synthesize magnetic gelatin-modified biochar with strong magnetic properties for the immobilization of arsenic (V) from water [52]. The maximum adsorption capacity for magnetic biochar was 45.8 mg/g, which was higher than that of the unmodified 17.5 mg/g, which also has a relatively high adsorption capacity value. The iron-impregnated corn straw biochar exhibited an excellent As^5+^ adsorption capacity of 6.80 mg/g compared to 0.017 mg/g for unmodified biochar [53]. The metal blending (six metal compounds—AlCl_3_, KCl, MgCl_2_, FeSO_4_, Cu(OH)_2_, Mg(OH)_2_ was doped prior to the pyrolysis of softwood shavings at 400 and 700 °C) of biochar was performed to study the adsorption behavior of anionic (arsenate and phosphate) pollutants [54]. AlCl_3_-blended biochar produced at 400 °C showed the highest arsenate adsorption (*Q*_m_ = 14.4 mg/g), whereas FeSO_4_-blended biochar pyrolyzed at 400 °C showed a lower value of maximum adsorption capacity (*Q*_m_ = 10.9 mg/g). Six different Fe-modified biochars were prepared for the simultaneous and individual adsorption of Cd^2+^ and As^5+^ oxyanion and compared with the pure pristine rice straw biochar [55]. These findings imply that the Fe modification of biochars, particularly those produced by pyrolyzing FeCl_3_ pre-soaked rice straw, significantly increased arsenic adsorption ability. The unconventional synthesis of magnetic biochar was introduced by Wang et al. [56]. A novel composite was synthesized by *Bacillus* sp. and K1-loaded onto Fe_3_O_4_ biochar for the simultaneous adsorption of two pollutants with adverse chemical behaviors (As^3+^; Cd^2+^). *Bacillus* sp. K1 treatment introduced new biosorption sites, including amine and hydroxyl groups, onto the composite surface, enhancing Cd^2+^ removal by 230% compared to raw magnetic biochar. In a binary system, maximum adsorption capacities of 25.04 mg/g and 4.58 mg/g were achieved for Cd^2+^ and As^3+^, respectively.

Due to the large active surface area, nano-zero-valent iron is another candidate for arsenic adsorption performance. In practical use, the preparation of zero-valent iron is not an easy task because the synthesis is carried out under strong reduction conditions. Moreover, according to Wang et al. [57], the aggregation effect appears to be due to small particle size. Therefore, nanosized zero-valent iron particles were loaded on the porous graphite oxide matrix with the aim of removing arsenite (*Q*_m_ = 38.8 mg/g) and arsenate (*Q*_m_ = 29 mg/g) from water. The efficiency of zero-valent iron was proven in the next work by Wang et al. [58]. Pine-derived biochar modified with nano-zero-valent iron shows excellent adsorption properties (*Q*_m_(arsenate) = 124.5 mg/g). In general, we have to say that immobilized zero-valent iron nanoparticles are an excellent adsorbent of inorganic arsenic with a sorption capacity of more than 100 mg/g. Table 1 displays the As^5+^ adsorption capabilities of several low-cost magnetic bioadsorbents.

In light of the numerous arsenic-contaminated sites in Slovakia (detailed in Section 1.2, 1.3 and Section 1.4) and the excellent adsorption efficiency of magnetic carbon biomaterials (see Section 1.5), we decided to test a synthesized magnetic bioadsorbent for arsenic removal from real wastewater. Furthermore, our prior studies [44,45,46,73] on the synthesis and testing of magnetic adsorbents in model aqueous solutions indicated that these biomaterials are effective for removing toxic oxyanions, cations, and dyes. Thus, the main goal was to verify and compare the adsorption performance of the magnetic biomaterial (MWchar-Mag) from both model and real wastewater, which, in addition to arsenic, contained many other metals. We investigated the influence of the initial adsorbent dosage, initial arsenic concentration, and pH and evaluated the adsorption kinetics.

## 2. Experimental Section

### 2.1. Preparation and Brief Characterization of the Selected Magnetic Adsorbent (MWchar-Mag)

Magnetic biochar was prepared as described by Zubrik et al. [44]. In the first step, biochar (MWchar) was made from wheat straw by microwave-assisted pyrolysis in a Panasonic NN-GD566M (Matsushita Electric Industrial Co., Ltd., Guangzhou, China) microwave oven for 6 min at a frequency of 2.45 GHz and 900 W. Subsequently, MWchar was modified by magnetic nanoparticles. The solution of Fe^3+^/Fe^2+^ ions (molar ratio 2:1) was mixed with MWchar in the mass ratio 1:1. The mixture was agitated for 60 min in water and degassed with N_2_ at pH 2. The solution was then treated with 24% ammonium hydroxide. The sample (MWchar-Mag) was precipitated for 60 min before being rinsed with deionized water to a neutral pH, filtered, and dried at 80 °C.

The MWchar-Mag adsorbent has been characterized by various methods (^57^Fe Mössbauer spectroscopy, X-ray diffraction, a low-temperature nitrogen adsorption method, and electron microscopy) in our previous work [44]. It is an adsorbent containing maghemite nanoparticles (with superparamagnetic properties). It is easy to remove it from the aqueous solution by an external magnetic field. Furthermore, by modifying the carbon matrix with magnetic nanoparticles, the adsorbent’s surface area also increased (S_BET_ = 139.1 m^2^/g), leading to higher adsorption efficiency. MWchar-Mag was tested for the adsorption of arsenic (As^5+^) and methylene blue under idealized model conditions (deionized water, optimal pH, and negligible ionic strength). The maximum monolayer adsorption capacity was determined to be 24.9 mg/g for arsenic oxyanion (at pH ~ 3.8) and 55.0 mg/g for methylene blue cation (at pH ~ 10.2).

### 2.2. Materials and Adsorption Procedure

All equilibrium adsorption tests were carried out under batch conditions in a rotary shaker at 30 rpm at room temperature. The metal concentration in the water solutions was measured using atomic absorption spectroscopy (AAS; Varian 240 RS/240 Z, Mulgrave, Australia), inductively coupled plasma mass spectrometry (ICP-MS; Agilent 7700 Series, Agilent Technologies, Tokyo, Japan) and ion chromatography (IC; Dionex ICS 5000, Thermo Scientific, Waltham, MA, USA). For low arsenic concentrations (<5 ppm), graphite furnace atomic absorption spectrometry (GFAAS) was performed (conditions: a matrix modifier of palladium solution plus ascorbic acid as a reducing agent; wavelength 193.7 nm; atomization temperature 2600 °C). The AAS and ICP-MS results were compared.

In the case of adsorption tests with a model aqueous solution, a stock solution of As^5+^ was prepared by dissolving AsHNa_2_O_4_·7H_2_O in deionized water. The adsorption test conditions for evaluating the effect of the initial adsorbent dosage were as follows: c_0_(As^5+^) = 9.15 mg/L; adsorbent dosage = 1–10 g/L; equilibrium adsorption time—24 h; and no pH adjustment. The adsorption isotherm experiment was conducted under the following conditions: c_0_(As^5+^) = 0.5–200 mg/L; c_MWchar-Mag_ = 2 g/L; adsorption time—24 h; and no pH adjustment. The adsorption kinetics were determined under the following experimental conditions: c_0_(As^5+^) = 9.4 mg/L; c_MWchar-Mag_ = 2 g/L; and no pH adjustment.

**Figure 1:**
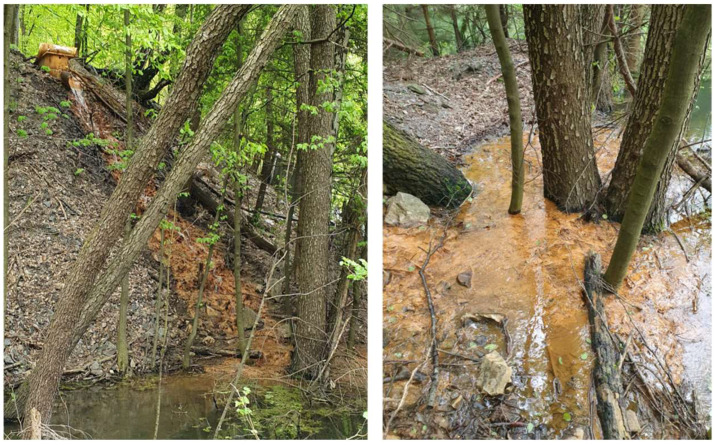
Outflow of mine water at the Hauser adit location.

For adsorption tests using real water, laboratory experiments were performed using water flowing from the Hauser adit (Figure 1). The experimental conditions for the adsorption tests were as follows: to evaluate the effect of the initial adsorbent dosage, the concentration of MWchar-Mag varied from 0.5 g/L to 10 g/L and the equilibrium adsorption time was set to 24 h (without pH adjustment); kinetics were performed with an initial adsorbent dosage of 1g/L (also without pH adjustment) and the effect of pH was studied over 24 h at the adsorbent dosage (MWchar-Mag) = 1 g/L and pH was changed with HNO_3_ and NaOH.

### 2.3. Data Analysis from Adsorption Experiments

Adsorption equilibrium data were evaluated using the Langmuir [74] and Freundlich [75] isotherm models, which describe monolayer adsorption on homogeneous surfaces and multilayer adsorption on heterogeneous surfaces, respectively. The evaluation of kinetic data was conducted using the pseudo-first-order (PFO) [76] and pseudo-second-order (PSO) [77] kinetic model (see Supplementary Materials, Table S1, for comprehensive information on all the models). A non-linear regression approach was used to assess the experimental data from the adsorption equilibrium studies and kinetics (PFO; PSO) using the Microsoft Excel 2010s Solver tool. It was proven that the non-linear forms of kinetic models [78], as well as the non-linear fitting of isotherm curves [79], are better ways to obtain the fitting parameter than the linear forms because of minimal errors during modeling (linearization).

## 3. Results

### 3.1. Characterization of Wastewater

Wastewater is flowing out of the old mine adit with a relatively low flow rate of 1.2 L/s, creating a pond below the backfilled mouth of the adit. Pit water in this locality is characterized by high arsenic and iron concentrations, which is evident in the rusty red color of precipitates in drained water and coatings on the surrounding rocks and soil. Arsenic concentrations vary from 0.20 to 0.36 mg/L [80,81,82] depending on the season and rainfall. Table 2 presents the chemical analysis of wastewater. Calcium, magnesium, sodium, potassium, iron, and manganese are the most abundant cations in the water. Anions include sulfates, chlorides, and arsenic. Thus, a high arsenic concentration (201.2 µg/L) was measured (20 times higher than the limited level in drinking water). The chemical composition of the water and the flow rate vary according to precipitation activity and seasonality. Notably, the pH of the wastewater from the Hauser adit is neutral (6.9).

### 3.2. Arsenic Removal from Model Aqueous Solution

**Figure 2:**
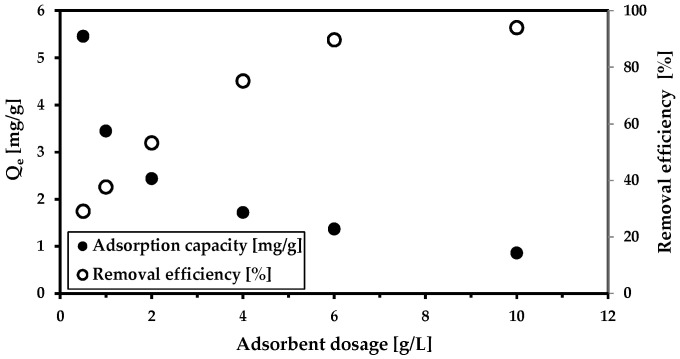
The effect of the adsorbent dosage on arsenic removal (c(As^5+^) = 9.15 mg/L; c_MWchar-Mag_ = 1–10 g/L). The adsorption was carried out in deionized water without pH adjustment (pH = 8.2).

We began with three basic adsorption studies on a model solution containing arsenate anion. We studied the effect of the adsorbent dosage and the effects of adsorbate concentration on solid magnetic composites and kinetics. In the first case (the adsorbent dosage influence), the removal efficiency increased with the increase in adsorbent dosage (Figure 2). There was no pH modification during the 24 h of adsorption, even though pH reached a slightly alkaline value (8.2). This increase in the pH is likely caused by the presence of the magnetic adsorbent (MWchar-Mag). Its isoelectric point is at 4.5 [44]. Above this isoelectric point, the adsorbent carries a negative charge, which attracts positively charged ions, thus decreasing their concentration in the solution and consequently raising the pH.

**Figure 3:**
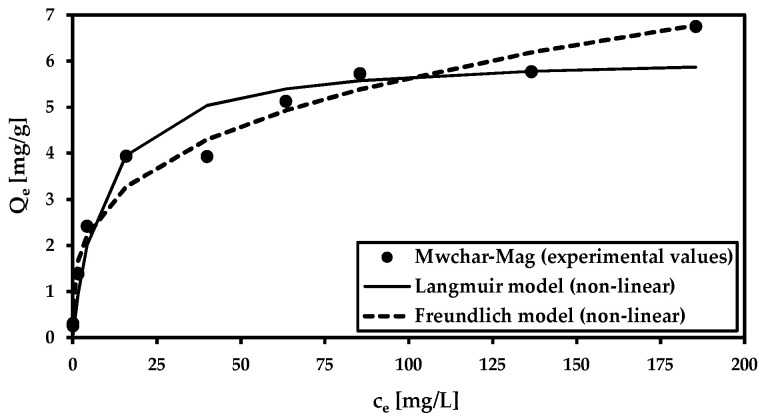
Adsorption isotherm for the model solution (c(As^5+^) = 0.5–200 mg/L; c_MWchar-Mag_ = 2 g/L; equilibrium pH = 8.2).

Adsorption isotherms (Figure 3) were analyzed using the Langmuir and Freundlich isotherm models (Table 3). The goal was used to calculate the maximum adsorption capacity at natural pH (pH was not adjusted), as well as to study the mechanism of the adsorption process. Both models are applicable to fit the data (r^2^ ≥ 0.95). In comparison to our earlier study [44], where the adsorption isotherm was performed at pH 3.8, and *Q*_m_ reached 24.9 mg/g, the maximum adsorption capacity decreased (*Q*_m_ = 6.2 mg/g at equilibrium pH 8.2).

**Figure 4:**
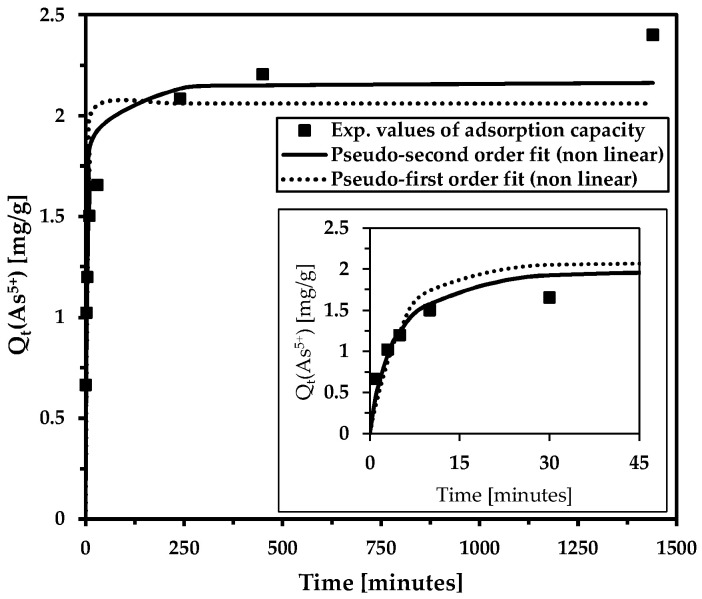
The influence of contact time on arsenic removal in the model solution (the inset shows the magnification of the initial time interval). Adsorption kinetics were evaluated using the non-linear pseudo-second-order and pseudo-first-order kinetic models. Basic conditions: c(As^5+^) = 9.4 mg/L; c_MWchar-Mag_ = 2 g/L; equilibrium pH = 8.2.

The adsorption rates of hazardous contaminants on adsorbents are crucial for future scaling and use. Adsorption kinetics are commonly characterized using pseudo-first-order and pseudo-second-order kinetic models. In general, the pseudo-second-order equation provides a better fit. This is consistent with our results (Figure 4, Table 3).

### 3.3. Adsorption of Arsenic from Real Water (Hauser Adit)

The effect of the adsorbent dosage, pH influence, and kinetics were investigated in order to remove arsenic from real water. The arsenic content in wastewater from Hauser adit was 201.2 µg/L. Although, in the case of the model test (Figure 2 and Figure 4), the concentration of arsenic was 46 times higher, i.e., ~9.2–9.6 mg/L), and it is important to note that arsenic competes with other ions during adsorption in real water samples. For this reason, manganese and iron removal efficiency was also monitored (see Supplementary Materials).

**Figure 5:**
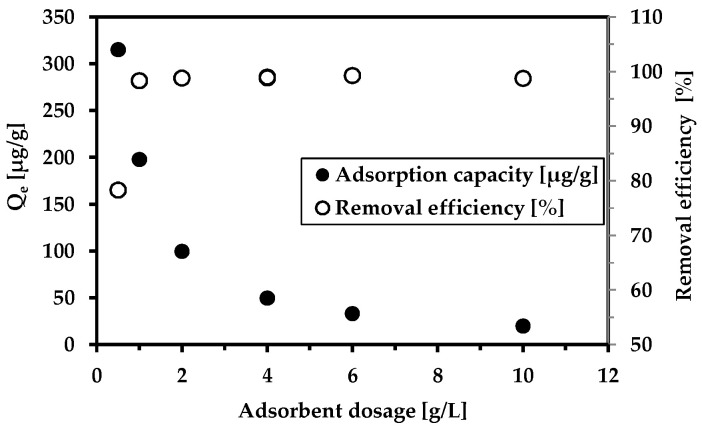
The effect of the adsorbent dosage on As^5+^ removal. Conditions: wastewater from Hauser adit (c(As^5+^) = 201.2 µg/L); adsorbent concentration (MWchar-Mag) = 0.5–10 g/L; equilibrium pH = 7.3 (no pH adjustments).

The effect of the adsorbent dosage on As^5+^ removal is highlighted in Figure 5. The findings verify the excellent performance of the magnetic adsorbent not only on the arsenic anion but also on manganese and iron. The removal efficiency reached 100% in the concentration range of 1–10 g/L. The pH monitored after adsorption was neutral, demonstrating the pH buffering effect of wastewater. Figure S1 shows the influence of the adsorbent dosage on Mn and Fe removal.

**Figure 6:**
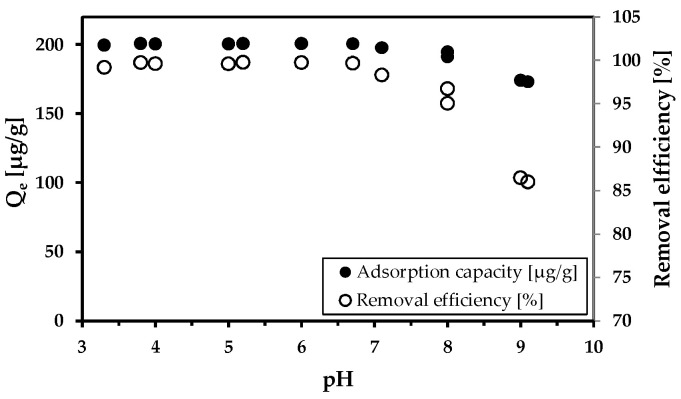
The effect of pH on As^5+^ removal. Conditions: wastewater from Hauser adit; adsorbent concentration (MWchar-Mag) = 1 g/L; pH change with HNO_3_ and NaOH.

The influence of pH on arsenic adsorption efficiency was also investigated (Figure 6). The effectiveness of removal was reduced when the pH increased above eight. In the acidic environment, removal efficacy was 100%. This demonstrates the perfect adsorption ability of MWchar-Mag. In Figure S2, the impact of pH on the elimination of Mn and Fe is also pointed out.

**Figure 7:**
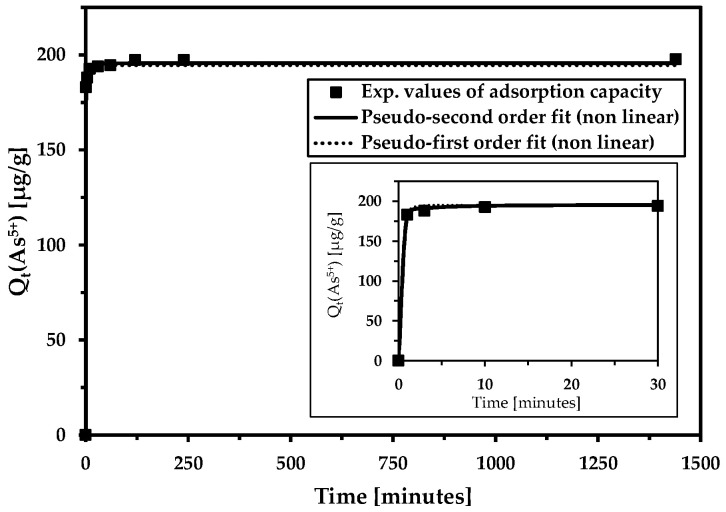
The influence of contact time on arsenic removal from wastewater (the inset shows the magnification of the initial time interval). Adsorption kinetics were evaluated using the non-linear pseudo-second-order and pseudo-first-order kinetic models. Basic conditions: c(As^5+^) = 201.2 µg/L; c_MWchar-Mag_ = 1 g/L; equilibrium pH = 7.3 (without pH adjustment).

The rate of arsenic removal from wastewater is extremely fast (Figure 7, Table 4). It is most likely caused by the relatively “low” arsenic content and the high adsorption capacity of the magnetic adsorbent. The other ions (cations and anions) detected in relatively high concentrations in the wastewater had minimal impact on the adsorption rate of the arsenic-to-magnetic adsorbent.

## 4. Discussion

**Figure 8:**
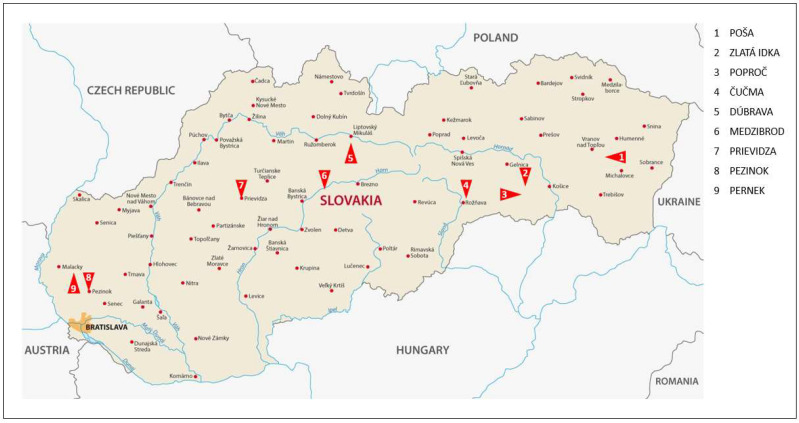
Contaminated areas with arsenic in Slovakia.

In the context of previous reviews (see Section 1.2, 1.3 and Section 1.4), there are many arsenic-contaminated areas in Slovakia. Four main localities were identified as follows (Figure 8, Table 5): the Spiš-Gemer region (Eastern Slovakia), the Little Carpathian (Western Slovakia), the Štiavnica and Kremnica Mountains (Southern Central Slovakia), and the Low Tatras Mts. (Central Slovakia). Mine wastewater from Hauser adit, which is the deepest horizontal adit in the entire Zlatá Idka ore district (Zlatá Idka village, Spiš-Gemer region of Eastern Slovakia), was chosen for our adsorption studies. The mine water discharged in this area was distinctive by elevated levels of arsenic due to the historical mining of As-bearing Au-Ag-Sb ores. The results of our analyses correlate with previously published data [83].

There are numerous studies based on the testing of magnetic adsorbents in model aqueous solutions. However, several examples of magnetic materials exist that are being used for the treatment of real water. For instance, Sirofloc technology [84], developed by CSIRO (The Commonwealth Scientific and Industrial Research Organisation), has applied recyclable magnetic particles to remove color and suspended matter from surface- and groundwater. Commercial products based on magnetic iron nanoparticles for arsenic removal from drinking water reviewed by Pradeep and Anshup [85] include AD33 (AdEdge Technologies, Inc., Atlanta, GA, USA) and ArsenX (SolrneteX, Inc., Marlborough, MA, USA). There are also commercial filters, such as the AD2710S Arsenic Reduction Water Filter Cartridge manufactured by AdEdge Water Technologies, LLC. (Atlanta, GA, USA), which, in addition to arsenic (As^3+^, As^5+^), also removes lead, cadmium, and chromium.

We realized comparative adsorption tests using both model and real wastewater conducted with MWchar-Mag, which has been previously identified as an effective arsenic adsorbent [44]. Its adsorption capacity reached a maximum at pH—3.8 (*Q*_m_ = 24.9 mg/g). The adsorption efficiency decreased with increasing pH, as confirmed by our experiments (Figure 3), where the adsorption was carried out under natural pH conditions (without pH adjustment). Thus, the value of maximum adsorption capacity is lower (*Q*_m_ = 6.2 mg/g, pH = 8.2) Note that model experiments were performed in ideal conditions (The adsorption of metal in deionized water without any additional influences such as ionic strength, competing ions, and ideal pH is set to reach the highest value of maximum adsorption capacity). The influence of pH on adsorption was further demonstrated by experiments conducted with mine water (see Figure 6). Excellent adsorption efficiency was achieved in a broad pH range. A decrease in adsorption capacity was observed at an alkaline pH.

The discussion regarding the sorption mechanism is based on our previous findings from testing magnetic materials for arsenite anions [46]. There were both physical and chemical mechanisms involved in the adsorption process [86]. Between pH 2.2 and 7.0, arsenate oxyanion dissociates in aqueous solutions, such as H_2_AsO_4_^−^, and inner-sphere complexation is the most commonly used process for arsenic removal. In our case, the HAsO_4_^2−^ form exists within the pH range of 7.0–11.5. It was proven [87] that the outer-sphere complexes are formed in alkaline environments. During adsorption in an alkaline environment, the adsorbent’s surface produces hydroxyl anions, and arsenic can be removed through ligand exchange (increasing the pH in the solution observed). Additionally, electrostatic interactions occur between the arsenic anions and the adsorbent surface. Because electrostatic attractions are weaker in alkaline environments, the maximum adsorption capacity, in our case, was found to be lower due to a repulsive electrostatic effect.

Rapid adsorption kinetics were observed in both the model aqueous solution and, notably, in real water samples containing other ions. This confirms the excellent sorption efficiency of magnetic adsorbent. The pseudo-second-order model generally predicts that the adsorption of the contaminant is primarily controlled by chemical reactions (ion exchange, coordination reactions, hydrogen bonding, and π–π interactions). The other pseudo-first-order model suggests that the pollutant is mainly adsorbed physically. Furthermore, the PFO suggests that the adsorption rate is controlled by adsorbate diffusion on the adsorbent surface. PSO kinetics are utilized for adsorption when it takes longer to fill the adsorption sites.

Materials derived from biomass frequently exhibit a porous structure with different cavities and surface locations. Pores and cavities increase the surface area available for adsorption. It provides more opportunities for metal-ion interaction. A biomass-based material has many functional groups [88], including hydroxyl, carboxyl, carbonyl, and amino groups (negatively charged). The magnetic phase provides a positive charge for the adsorption of oxyanions. These promote the efficiency of magnetic biomaterials in wastewater treatment applications. We assume that arsenic adsorption to magnetic biochar can be controlled by a variety of adsorption mechanisms, such as physical adsorption (van der Waals forces), chemisorption, electrostatic interactions, (intra-particle) diffusion, hydrogen bonding, complexation, and ion exchange, etc. Moreover, our results indicated that the material composed of organic carbon and inorganic iron oxides is able to absorb both anionic and cationic metals/metalloids (see Supplementary Materials, Figures S1 and S2).

## 5. Conclusions

In this paper, we first reviewed arsenic contamination issues in Slovakia because arsenic is a persistent mobile pollutant with a high degree of toxicity. The mining, mineral processing, ore roasting, metallurgy, metal winning, and combustion of fossil fuels in the past have all contributed to the contamination of water and soil with arsenic. The Spiš-Gemer region in Eastern Slovakia, the Little Carpathian region in Western Slovakia, the Štiavnica and Kremnica Mountains in Southern Central Slovakia, and the Low Tatras Mountains in Central Slovakia were found to be the four primary localities contaminated by arsenic. Carbon biochars (made from waste biomass) that have been modified with iron (nano) particles, such as magnetite, hematite, zerovalent iron, and maghemite, show potential as solutions for removing arsenic from wastewater. We decided to evaluate magnetic carbon biochar (MWchar-Mag) as an arsenic adsorbent from both real and mine wastewater (from Hauser adit, the Spiš-Gemer region in Eastern Slovakia) and model solutions. The maximal adsorption capacity for the model aqueous solution was determined to be 6.2 mg/g at pH 8.2 (without pH adjustment). The results obtained from the model tests (the fast kinetics and high value of *Q*_m_) were confirmed during adsorption with real wastewater. The adsorbent dosage, pH effect investigations, and kinetic testing confirm 100% arsenic removal effectiveness at pH~3–7, with quick kinetics in the mine water effluent and the concentration of As^5+^ at about 0.2 mg/L.

To sum up, the use of organic waste material/natural bioadsorbents from local sources for the preparation of advanced carbonaceous adsorbents with low production costs, non-toxicity, long adsorbent life, favorable physical properties (such as ease of handling), reusability, and minimal reactivity offer a viable solution in many regions, e.g., in economically developing countries and regions in industrial transition to resolve environmental issues.

## Figures and Tables

**Table 1:** Arsenate maximum sorption capacities (*Q*_m_) for various magnetic biochars.

Adorption of As^5+^	*Q*_m_ (mg/g)	Reference
Fe/Ca-rich biochar (paper mill sludge)	22.8	[59]
Pristine pinewood biochars/zero-valent Fe	124.5	[58]
Magnetic chitosan biochar	17.9	[60]
Magnetic gelatin-modified biochar (chestnut shell)	45.8	[52]
Magnetic biochar (spent coffee ground)	12.1–12.6	[50]
Magnetic porous carbonaceous material (tea waste)	38.0	[47]
Immobilized Fe_3_O_4_/bone char nanocomposite	0.1	[61]
Magnetic biochar (wheat straw/ferrofluid)	25.6	[45]
Iron-impregnated corn straw biochar	6.8	[53]
Zero-valent iron biochar (switchgrass)	7.9	[62]
Zero-valent iron biochar (red oak)	15.6	[62]
Magnetic char (coal/ferrofluid)	19.9	[46]
Iron-modified rice straw biochar	26.9	[63]
Al-blended softwood biochar	14.4	[54]
Fe-blended softwood biochar	10.9	[54]
Fe oxide nanoneedle biochar (cotton fibers)	8.13	[64]
Humic acid/Fe-Mn oxide-loaded biochar (rice husk)	35.6	[65]
Activated bamboo biochar/Fe_3_O_4_	85	[66]
Bamboo biochar/Fe_3_O_4_	90	[66]
Fe-modified biochars (Poplar tree)	87.3–121.6	[67]
Zero-valent iron immobilized on cotton fabric	108.7	[68]
Nano-zero valent iron and sewage sludge	11.3	[69]
Carbon/Al_2_O_3_/nano-zero-valent iron material	20.2	[70]
Magnetic eucalyptus wood charcoal	0.021	[71]
Lignin biochar/FeO_X_	6.8	[48]
Magnetic biochar (wheat straw/maghemite)	24.9	[44]
Zero-valent iron/biochar composite (bamboo)	127.2	[72]

**Table 2:** Chemical analysis of wastewater from Hauser adit. Sampling 22 April 2024.

	pH	Mn [µg/L]	Fe [µg/L]	Ba [µg/L]	Sb [µg/L]	As [µg/L]	Ni [µg/L]
Method	-	AAS	AAS	ICP-MS	ICP-MS	ICP-MS	ICP-MS
Value	6.9	425	1428	2.5	0.6	201.2	1.2
	Zn [µg/L]	Cu [µg/L]	Cd [µg/L]	Pb [µg/L]	Cr [µg/L]	Co [µg/L]	Li [µg/L]
Method	ICP-MS	ICP-MS	ICP-MS	ICP-MS	ICP-MS	ICP-MS	IC
Value	21.7	1.6	<0.1	0.8	0.2	0.7	12.4
	Na [µg/L]	NH_4_^+^ [µg/L]	K [µg/L]	Mg [µg/L]	Ca [µg/L]		
Method	IC	IC	IC	IC	IC		
Value	5334	34.6	864	12,140	21,880		
Element	F^−^ [µg/L]	Cl^−^ [µg/L]	SO_4_^2−^ [µg/L]				
Method	IC	IC	IC				
Value	119	1033	31,334				

Abbreviations: IC, ion chromatography; AAS, atomic absorption spectroscopy; ICP-MS, inductively coupled plasma mass spectrometry.

**Table 3:** Calculated values from the kinetics and adsorption isotherms obtained using a non-linear fitting method in the case of the arsenic model solution.

Langmuir Isotherm Model		Freundlich Isotherm Model		Pseudo-Second-Order Kinetic Model		Pseudo-First-Order Kinetic Model	
*Q*_m_ [mg/g]	6.2	*K_F_* [L/g]	1.44	*q*_e experimental_ [mg/g]	2.41	*q*_e experimental_ [mg/g]	2.41
*b* [L/mg]	0.13	*N*	3.37	*q*_e teoretic_ [mg/g]	2.17	*q*_e teoretic_ [mg/g]	2.06
*R* ^2^	0.95	*R* ^2^	0.97	*k*_2_ [g/mg.min]	0.122	*k*_1_ [1/min]	0.186
				*h*_2_ [mg/g.min]	0.575	*h*_1_ [mg/g.min]	0.382
				*R* ^2^	0.961	*R* ^2^	0.903

**Table 4:** Calculated values from the kinetics obtained using a non-linear fitting method in the case of real wastewater.

Pseudo-Second-Order Kinetic Model		Pseudo-First-Order Kinetic Model	
*q*_e experimental_ [µg/g]	198.6	*q*_e experimental_ [µg/g]	198.6
*q*_e teoretic_ [µg/g]	195.8	*q*_e teoretic_ [µg/g]	194.6
*k*_2_ [g/µg min]	0.0659	*k*_1_ [1/min]	2.812
*h*_2_ [µg/g min]	2526	*h*_1_ [µg/g min]	547.2
*R* ^2^	0.999	*R* ^2^	0.998

**Table 5:** The localities of Slovakia markedly contaminated with arsenic.

Town/Village	Region	Pollution and Comments
Poša (1)	Eastern Slovakia	Chemical industry
Zlatá Idka (2)	Eastern Slovakia	As bearing Au–Ag–Sb ores
Poproč (3)	Eastern Slovakia	Tailings from the flotation of sulfide ores; As/Sb contamination
Čučma (4)	Eastern Slovakia	Old adits; Sb and As contamination
Dúbrava (5)	Central Slovakia	Old mine adits; Sb mining in the past; Sb/As contamination
Medzibrod (6)	Central Slovakia	Sulfide ore extraction; Sb/As contamination
Prievidza (7)	Central Slovakia	Coal mining and power plant; As in fly ash
Pezinok(8)	Southwestern Slovakia	Adit outflows; As and Sb contamination
Pernek (9)	Southwestern Slovakia	Deposits; Sb and As contamination

## Data Availability

The original contributions presented in the study are included in the article/Supplementary Material, further inquiries can be directed to the corresponding authors.

## Supplementary Materials

The following supporting information can be downloaded at: https://www.mdpi.com/article/10.3390/ma18020289/s1. Table S1. Models for evaluation of the adsorption processes. Figure S1. The effect of adsorbent dose on Mn (left) and Fe (right) removal. Conditions: wastewater from Hauser adit (c_0_((Fe) = 1428 µg/L); c_0_((Mn) = 425 µg/L)); adsorbent concentration (MWchar-Mag) = 0.5–10 g/L; 24 h, pH = 7.3 (no pH adjustments). Figure S2. Influence of pH on Mn (left) and Fe (right) removal. Conditions: wastewater from Hauser adit (c_0_((Fe) = 1428 µg/L); c_0_((Mn) = 425 µg/L)); adsorbent concentration (MWchar-Mag) = 1 g/L; 24 h, RT, pH change with HNO_3_ and NaOH.
