# Supplementary material for: Arsenic in Slovakia: Pollution Issues and the Potential of Magnetic Carbon Biomaterials for Wastewater Treatment
Source: Materials (Basel). 2025 Jan 10;18(2):289. doi: 10.3390/ma18020289 (PMC11767112; doi:10.3390/ma18020289)

Supplementary material

Arsenic in Slovakia: Pollution Issues and the Potential of Magnetic Carbon Biomaterials for Wastewater Treatment

Anton Zubrik [*], Eva Mačingová, Slavomír Hredzák and Marek Matik

Institute of Geotechnics, Slovak Academy of Sciences, Watsonova 45, SK-04001 Košice, Slovakia

[*] Corresponding author: Anton Zubrik

Tel.: +421 557922630

e-mail address: zubant@saske.sk

Postal address: Institute of Geotechnics, Slovak Academy of Sciences, Watsonova 45, 04001 Košice, Slovakia

Comment to supplementary material:

Our supplementary material contains Table S1, which presents the equations used to calculate the parameters for evaluating adsorption isotherms, as well as the parameters characterizing adsorption kinetics. While this study primarily focused on the removal of arsenic, the adsorption of iron and manganese from real wastewater was also observed. Figures S1 and S2 illustrate the effects of adsorbent dosage and pH on the removal of iron and manganese.

Content

Table S1 Page 2

Figure S1, Figure S2 Page 3

Table S1. Models for evaluation of the adsorption processes.

Adsorption/kinetic model	Equation	Comments
Langmuir isotherm	$q_e = Q_m \frac{bC_e}{1+bC_e}$	q_e is equilibrium adsorption capacity (mg/g), Q_m is the maximum adsorption capacity (mg/g), C_e is the equilibrium metal concentration, and b is a Langmuir constant characterizing the affinity between the adsorbed molecule and the adsorbent (L/mg).
Freundlich isotherm	$q_e = K_F C_e^{\frac{1}{n}}$	K_F (L/g) and n are constants of the isotherm. Q_m, b, K_F, and n were determined from the experimental data using the linearized forms of the above equations.
Pseudo-first order kinetic model (PFO)	$\frac{dq_t}{dt} = k_1.(q_e - q_t)$	k_1 is the adsorption rate constant of the first order (min^{-1}); q_e is equilibrium adsorption capacity (mg/g); q_t is the uptake capacity at any time (mg/g).
Integrated form of PFO	$q_t = q_e[1 - \exp(-k_1 t)]$	Integration at defined boundary conditions (t = 0, q_t = 0 and t = t, qe = q_t).<br>Initial sorption/desorption rate h_1 (mg/g.min) is defined as:<br>$h_1 = q_e.k_1$
Pseudo-second order kinetic model (PSO)	$\frac{dq_t}{dt} = k_2.(q_e - q_t)^2$	k_2 is adsorption rate constant of the second order (g/mg.min). q_e (mg/g) is the adsorbate equilibrium amount and q_t is the uptake capacity at any time (minutes).
Integrated form of PSO	$q_t = \frac{k_2.q_e{}^2.t}{1+k_2.q_e.t}$	Integration at defined boundary conditions (t = 0, q_t = 0 and t = t, q_e = q_t).<br>Initial adsorption/desorption rate h_2 (mg/g.min) is defined as:<br>$h_2 = k_2.q_e^2$

Figure S1. The effect of adsorbent dose on Mn (left) and Fe (right) removal. Conditions: wastewater from Hauser adit (c_0((Fe) = 1428 µg/L; c_0((Mn) = 425 µg/L); adsorbent concentration (MWchar-Mag) = 0.5-10 g/L; 24 h, pH = 7.3 (no pH adjustments).

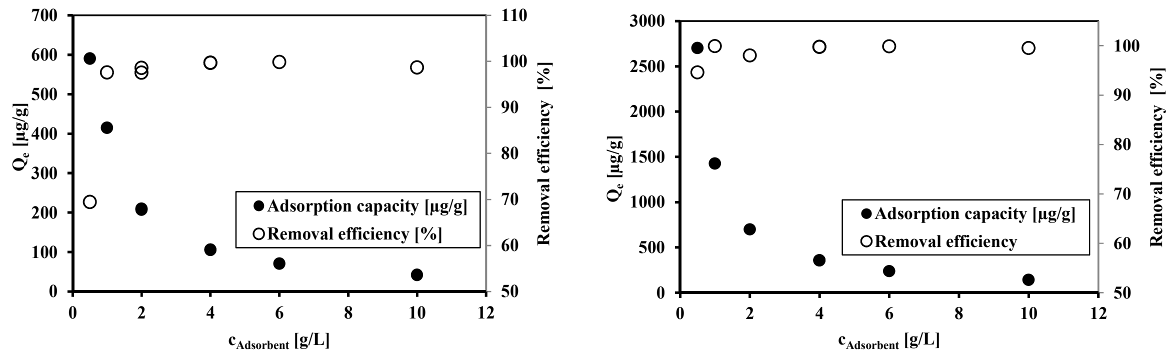

Figure S2. Influence of pH on Mn (left) and Fe (right) removal. Conditions: wastewater from Hauser adit (c_0((Fe) = 1428 µg/L; c_0((Mn) = 425 µg/L); adsorbent concentration (MWchar-Mag) = 1 g/L; 24 h, RT, pH change with HNO_3 and NaOH.

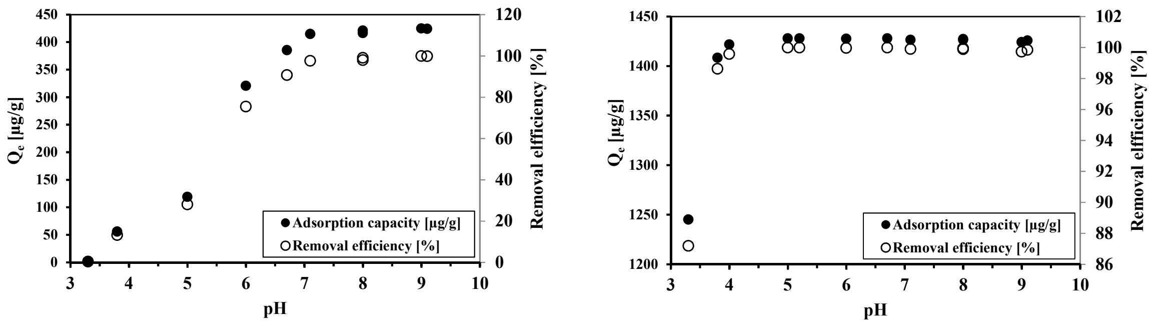

Supplement: Supplementary file 1 [file materials-18-00289-s001.zip › materials-3395825-supplementary.pdf]
